# Biguanide- and Oligo(Ethylene Glycol)-Functionalized Poly(3,4-Ethylenedioxythiophene): Electroactive, Antimicrobial, and Antifouling Surface Coatings

**DOI:** 10.3389/fchem.2022.955260

**Published:** 2022-08-05

**Authors:** Hailemichael Ayalew, Syed Atif Ali, Jia-Wei She, Hsiao-hua Yu

**Affiliations:** ^1^ Smart Organic Materials Laboratory, Institute of Chemistry, Academia Sinica, Taipei, Taiwan; ^2^ Taiwan International Graduate Program (TIGP), Sustainable Chemical Science and Technology (SCST), Academia Sinica, Taipei, Taiwan; ^3^ Department of Applied Chemistry, National Yang Ming Chiao Tung University, Hsinchu, Taiwan

**Keywords:** Poly(3,4-ethylenedioxythiophene), electropolymerization, biguanide, antimicrobial, antifouling

## Abstract

The challenge of infectious diseases remains a critical concern to the global public health. Recently, it is common to encounter touch-screen electronic devices everywhere to access services. The surface of such devices may easily get contaminated by an infected person, which leads to transmission of infectious diseases between individuals. Moreover, the challenge is complicated by surgical infections from implantable biomedical devices. Such problems can be minimized by the use of long-term active antimicrobial surface coatings. We present herein the preparation of novel electroactive antimicrobial surface coatings through the covalent attachment of the biguanide moiety onto 3,4-ethylenedioxythiophene (EDOT). The biguanide-functionalized EDOT (EDOT-BG) was thus electropolymerized on different substrates to give the corresponding poly(EDOT-BG) polymer. The poly(EDOT-BG) polymer showed an excellent bactericidal efficiency (∼92% bacterial death) and excellent biocompatibility with mammalian cells. Furthermore, the antimicrobial EDOT-BG was electro-copolymerized with antifouling tetra ethylene glycol functionalized-EDOT (EDOT-EG4) to give a multifunctional poly(EDOT-EG4-*co*-EDOT-BG) copolymer. The poly(EDOT-EG4-*co*-EDOT-BG) copolymer showed excellent resistance to protein adsorption and mammalian/bacterial cell binding without losing its bactericidal efficiency. These novel materials can be applied to domestic and bioelectronic devices to minimize infectious diseases.

## Graphical Abstract

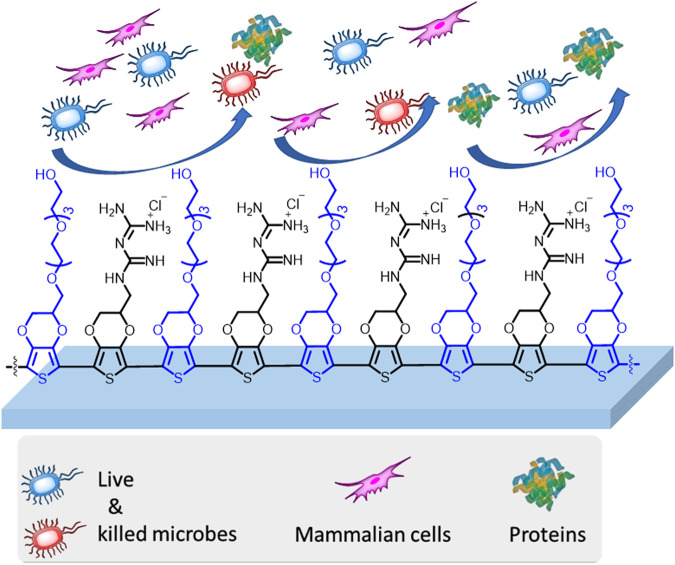

## 1 Introduction

Developing functional surface coatings for domestic and biomedical devices is imperative to enhance their durability and safer use. Functional surface coatings such as self-cleaning (Zhu D. et al., 2014), electrochromic (Gunbas and Toppare, 2012), fire-retardant (Weil, 2011), antifogging (Wang et al., 2015), hydrophobic (Park et al., 2012), antimicrobial (Siedenbiedel and Tiller, 2012), and antifouling (Goda and Miyahara, 2019) have been extensively studied. Among the coating materials, antimicrobial surface coatings attract significant attention due to rising concerns about microbial infections including the current Covid-19 (Rakowska et al., 2021) infection. Previously, various antimicrobial surface coatings with different killing mechanisms (Siedenbiedel and Tiller, 2012) such as biocide-releasing (Stasko and Schoenfisch, 2006; Rosenberg et al., 2008; Coneski et al., 2010), contact-active (Tiller et al., 2001; Tiller et al., 2002), or repelling (Ho et al., 2004) have been reported for textiles (Dhende et al., 2011), glasses (Zhang et al., 2010), and biomedical devices (Hasan et al., 2013; Zander and Becker, 2018). Apparently, the activity of biocide-releasing surfaces declines through time as the concentration of biocides to be released decreases and may not be enough to kill the microorganisms. In addition, such systems could have harmful consequences to the surrounding environment due to the toxicity of released biocides. On the other hand, the antimicrobial activity of contact-active surfaces will be affected by the deposition of dead microorganisms or other fouling agents in the long run. Therefore, the development of long-term active, biocide-free, and antifouling surface coatings is indispensable.

For instance, antimicrobial coatings can be prepared by covalent linkage of the antimicrobial moieties onto the available materials such as polymers. In this manner, cationic polymeric materials functionalized with quaternary ammonium and peptide groups have been prepared, and their antimicrobial activities were studied extensively (Tew et al., 2002; Gabriel et al., 2008; Tew et al., 2010; Grace et al., 2016). Specifically, guanidine and biguanide derivatives such as poly(hexamethylene biguanide hydrochloride) (PHMB) are prominent antimicrobial disinfectants so far (Ikeda et al., 1984; Budhathoki-Uprety et al., 2012; Bottcher et al., 2013; Zhi et al., 2017). For example, a report by Novak’s group demonstrated moderate to significant antibacterial activity of guanidinium-functionalized polycarbodiimides against some Gram-positive and Gram-negative bacteria (Budhathoki-Uprety et al., 2012). Similarly, Clardy’s group prepared norspermidine (natural biofilm disrupter) to mimic antimicrobial guanidine and biguanide-containing compounds that showed up to 20-fold increased potency in preventing biofilm formation and breaking down of existing biofilms (Bottcher et al., 2013). Further study by Zhi et al. demonstrated the preparation of dual-purpose surface coatings through the conjugation of antimicrobial PHMB with antifouling allyloxy polyethylene glycol that showed broad-spectrum antimicrobial activity and potent antibiofilm properties (Zhi et al., 2017).

Likewise, electrically conducting polymers have been explored in the preparation of functional surface coatings due to their responses to electrical stimuli, flexibility compared to metal counterparts, and easy modification of the side chains for specific applications (Ayalew et al., 2019). In addition, the properties of conducting polymer materials can easily be tuned by copolymerization of different monomers each with specific properties to get an intermediate/new property compared to the individual polymers (Zhao et al., 2013; Malmstrom et al., 2017). In this regard, poly(3,4-ethylenedioxythiophene) (PEDOT) is one of the most studied conducting polymers in the preparation of antimicrobial and/or antifouling surface coatings with the ease of covalent linkage of functional moieties on the monomer and well-established electropolymerization techniques (Reynoso et al., 2021). Electropolymerization helps to deposit the polymers directly on a substrate of interest. A good example is a zwitterionic sulfobetaine-functionalized-PEDOT (PSBEDOT) studied by Cao et al. that showed switchable antifouling and antimicrobial properties under different applied potentials. The cationic PSBEDOT surface showed good antimicrobial activity, while the zwitterionic PSBEDOT surface was highly resistant to cell attachment (Cao et al., 2016). However, such switchable materials require control of the surface potential through electrochemical techniques that could be difficult to control from a remote distance and in complex environments such as in implanted bioelectronic devices.

Therefore, this study is intended to develop a new electrically conductive dual-purpose surface coating that can kill approaching microbes and less adhesive to biofilms or proteins and cells. Hence, biguanide was chosen as the antimicrobial moiety and covalently attached to 3,4-ethylenedixoxythiophene (EDOT) to get the biguanide-functionalized EDOT (EDOT-BG) monomer. EDOT-BG was then electrodeposited on indium-tin-oxide coated glass slides (ITO-glass) to make the corresponding biguanide-functionalized PEDOT (poly(EDOT-BG)) polymer. The antimicrobial activity and the biocompatibility of poly(EDOT-BG) were investigated using *E. coli* and human embryonic kidney (HEK-293T) cells, respectively, as models. Previous studies by our group and others proved the antifouling properties of oligo ethylene glycol-functionalized EDOTs such as EDOT-EG4 (Zhao et al., 2013). Similarly, herein, EDOT-BG was electrochemically copolymerized with EDOT-EG4 to prepare a multifunctional poly(EDOT-EG4-*co*-EDOT-BG) copolymer. The simultaneous antimicrobial and antifouling properties of the poly(EDOT-EG4-*co*-EDOT-BG) copolymer were investigated from bactericidal and protein or cell adhesion studies. The electrodeposited polymers were characterized by scanning electron microscopy (SEM), energy-dispersive X-ray (EDX), X-ray photoelectron spectroscopy (XPS), and cyclic voltammetry (CV) techniques. We believe that these biguanide-functionalized PEDOT materials could have a potential application in the preparation of long-term active antimicrobial and antifouling surface coatings for domestic and biomedical devices.

## 2 Materials and Methods

### 2.1 Chemicals and Measurements

All the chemicals were of reagent grade and used without further purification. All reactions were carried out under an N_2_ atmosphere using anhydrous solvents.

^1^H and ^13^C NMR spectra were recorded with Bruker AVIII-400 spectrometers, and chemical shifts were measured in δ (ppm) with residual solvent peaks as internal standards. UV–visible (UV/Vis) spectra were measured on an Agilent Technologies Cary 8454 UV-V is spectrophotometer. The mass spectrum was obtained on a TOF-MS spectrometer. The morphology of the electrodeposited polymers on ITO-glass slides was recorded using a field-emission scanning electron microscope (FE-SEM, ULTRA PLUS). Electrochemical polymerizations and electrochemical studies were performed using an Autolab potentiostat (PGSTAT128N, ECO CHEMIE BV, the Netherlands). The XPS spectra were acquired with a PHI 5000 VersaProbe (ULVAC-PHI, Chigasaki, Japan) spectrometer with a 24.7 W micro focused Al kα X-ray source and a take-off angle of photoelectron at 45°. The fluorescence microscopy images were recorded with a Nikon–ECLIPS Ni-E microscope (Nikon Corporation, Tokyo, Japan) and Olympus IX81 fluorescence microscope (Japan).

### 2.2 Synthesis of the Monomer and Polymers

#### 2.2.1 Synthesis of Biguanide Hydrochloride Bearing 3,4-Ethylendioxythiophene

First, amino-functionalized 3,4-ethylendioxythiophene (EDOT-NH_2_) was prepared from the hydroxymethyl-functionalized EDOT (EDOT-OH) starting material in a few steps. The detailed synthesis procedure for EDOT-NH_2_ is presented in the Supplementary Information. The biguanide hydrochloride-functionalized EDOT (EDOT-BG) was then synthesized from EDOT-NH_2_ as follows: EDOT-NH_2_ (1.0 g, 5.85 mmol) was added to a 100 ml two-neck round-bottom flask, backfilled with N_2_ gas, and dissolved in dimethyl formamide (DMF) (1.5 ml). To the above solution, HCl (37%, 0.49 ml) was added dropwise via a syringe. A white solid was formed, and it was dissolved by increasing the temperature to 60°C. Once dissolved, the mixture was cooled to room temperature and dicyandiamide (0.54 g, 6.43 mmol) dissolved in DMF (1.5 ml) was added via a syringe, followed by refluxing the contents at 85°C for 5 h under an N_2_ atmosphere. The reaction mixture was cooled to room temperature, and excess ethyl acetate was added. A colorless solid was precipitated, filtered, and washed with ethyl acetate (3x) to yield the corresponding EDOT-BG product (1.7 g, 83%). ^1^H NMR (400 MHz, DMSO-d_6_) δ: 8.73 (b, s, 4H), 6.77 (s, 1H), 6.64 (dd, 2H, J = 5.3, 3.6 Hz), 4.52 (b, s, 1H), 4.37–4.34 (m, 1H), 4.04 (dd, 1H, J = 11.8, 7.3 Hz), 3.19 (dd, 1H, J = 13.5, 3.4 Hz), 3.02 (dd, 1H, J = 13.3, 8.1 Hz). ^13^C NMR (100 MHz, DMSO-d_6_) δ: 155.7, 154.5, 140.9, 140.2, 100.5,100.2, 70.5, 65.3, 38.5. HR (ESI-MS) m/z: [M + H]^+^ Calcd for C_9_H_13_N_5_O_2_S: 256.0868; found: 256.0866.

#### 2.2.2 Electropolymerization and Characterization of the Polymers

**FIGURE 1:**
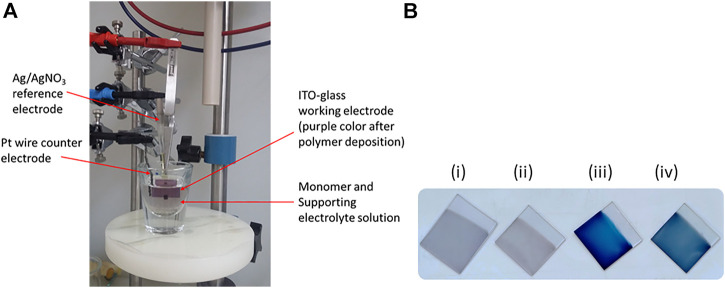
**(A)** Three-electrode system electropolymerization cell set-up [the purple color of the working electrode (ITO-glass) shows the electrodeposited polymer film] and **(B)** photo of the polymer films of poly(EDOT-BG) (i), poly(EDOT-BG-*co*-EDOT-EG4) (ii), PEDOT (iii), and poly(EDOT-*co*-EDOT-BG) (iv) electrodeposited on ITO-glass slides.

The poly(EDOT-BG) homopolymer and copolymers with either unfunctionalized EDOT or EDOT-EG4 were deposited on ITO-glass slides through the electropolymerization technique. The monomer solutions were prepared by dissolving the appropriate amount of monomer/s in MeCN to give a final concentration of 10 mM. To enhance the solubility of EDOT-BG, conc. H_2_SO_4_ (3%, v/v) was added to the MeCN solution. In addition, the sodium dodecyl sulfate (SDS) surfactant (20 mM) was added to enhance the polymer film stability to be used in aqueous solvents. A three-electrode system electrochemical cell with a Ag/AgNO_3_ reference electrode, a Pt wire counter electrode, and ITO-glass slides as a working electrode was employed. The polymerization was performed by applying a constant potential of 1.0 V for 60 s in the presence of LiClO_4_ electrolyte (0.1 mM). The polymer films were rinsed with water and MeCN to remove monomer and electrolyte residues and dried with nitrogen blowing (Figures 1A,B). The polymer films were characterized with SEM, UV/Vis spectroscopy, XPS, and CV.

### 2.3 Cell Viability Studies on Biguanide-Functionalized PEDOT Surfaces

For biocompatibility studies of biguanide-functionalized PEDOTs, human embryonic kidney (HEK-293T) cell lines were used. The polymer-coated ITO-glass slides (1 × 1 cm) were sterilized with 70% (v/v) ethanol for 20 min, followed by rinsing with phosphate-buffered saline (PBS) (Uni-Region Bio-Tech, Taiwan) three times. The slides were then placed separately into a 12-well cell culture plate, and freshly prepared HEK-293T cells were seeded onto each slide at a density of 2 × 10^4^ cells/cm^2^. The cells were incubated with complete Dulbecco’s modified Eagle’s medium (DMEM, Life technologies, United States) at 37°C for 48 h in 5% CO_2_. The cells were then stained with a live/dead cell double staining kit (Life technologies, United States) according to the manufacturer’s guidelines, and thus, the fluorescent signal was obtained using fluorescence microscopy (IX83, Olympus, Japan). The live (green) and dead (red) cells were counted, and the data are presented in mean ± standard deviation (SD) with three replicates. In addition to the live/dead cell viability assay, the biocompatibility of the prepared materials was performed with the MTT viability assay, and the detailed procedure is mentioned in the Supplementary Information.

### 2.4 Antimicrobial Property

The antimicrobial activity study was performed using *Escherichia coli* (*E. coli*) bacteria as a model. The detailed bacterial sample preparation and bactericidal activity study procedures are stated in the Supplementary Information. Briefly, the polymer-coated ITO-glass slides (1 × 1 cm) were placed in 12-well cell culture plates and sterilized with 70% ethanol for 20 min. The *E. coli* sample (1 × 10^6^ cells/mL) in PBS was seeded on each slide and incubated for 2 h at 37°C. After the incubation, the samples were washed with PBS and the bactericidal activities for all the samples were analyzed by OD (600 nm) measurements and live/dead cell staining assay methods.

### 2.5 Antifouling Properties of poly(EDOT-EG4-*co*-EDOT-BG): Protein Adsorption and Cell Adhesion

*Protein adsorption*. In order to study protein adsorption properties, quartz crystal microbalance (QCM) measurement was carried out on a Q-Sense AB system (Biolin Scientific, QE401-F1521, Sweden) at 25°C. The poly(EDOT-BG) and poly(EDOT-EG4-*co*-EDOT-BG) polymers were electrodeposited on gold QSX 301 sensor crystals. Each crystal was fixed in the measurement chamber. A baseline was established by injecting 1X PBS buffer (pH 7.2) through the chamber at a rate of 50 μL/min. Following a baseline stabilization, the bovine serum albumin (BSA) protein solution (1 mg/ml) was injected at a rate of 50 μL/min for about 20 min. Afterward, PBS buffer was injected to remove loosely bound protein from the polymer surface until equilibrium was reached. Finally, a change in frequency (Δf) between the PBS background baseline and the equilibrium baseline was calculated to determine the antifouling properties of the polymers. The QCM measurements were performed at least twice to confirm the reproducibility.

*Cell Adhesion Tests*. The antifouling property of the copolymer was demonstrated using mammalian and bacterial cells. For mammalian cell adhesion studies, HEK-293T cells were used as a model. First, the polymer-coated ITO-glass slides (1 × 1 cm) were sterilized with 70% alcohol and placed in 12-well cell culture plates. The cells were then seeded on each sample at a density of 2 × 10^4^/ml. The cells were incubated at 37°C with 5% CO_2_ for 48 h. Finally, the slides were washed with PBS gently, and the cells were stained with a live/dead cell staining kit (Life technologies, United States) according to the manufacturer’s guidelines. The fluorescence signal of the attached cells was observed under fluorescence microscopy (IX83, Olympus, Japan). The antifouling property of poly(EDOT-EG4-*co*-EDOT-BG) was determined by comparing the cell density data with PEDOT, poly(EDOT-*co*-EDOT-BG), and poly(EDOT-BG) polymers. The results were determined in mean ± SD.

Similarly, for antifouling studies on the bacterial sample, the polymer-coated ITO-glass slides were sterilized and placed in cell culture plates. 50 µL of *E. coli* at a concentration of 1 × 10^6^ cells/mL was pipetted onto each polymer-coated sample and incubated for 2 h at room temperature. The slides were then washed with sterile PBS, and the surface-attached bacteria were stained with a live/dead bacterial viability kit (Thermo Fisher Scientific, Cat. L7007) for 15 min in the dark. After gently rinsing with sterile water and drying in the air, the surface-attached bacteria were examined using a Nikon–ECLIPS Ni-E fluorescence microscope (Nikon Corporation, Tokyo, Japan). Three images were chosen randomly for each surface with three replicates, and the relative number of living (green) versus dead (red or yellow) bacteria was counted using ImageJ software.

## 3 Results and Discussion

### 3.1 Synthesis of the EDOT-BG Monomer and Polymers

**SCHEME 1:**
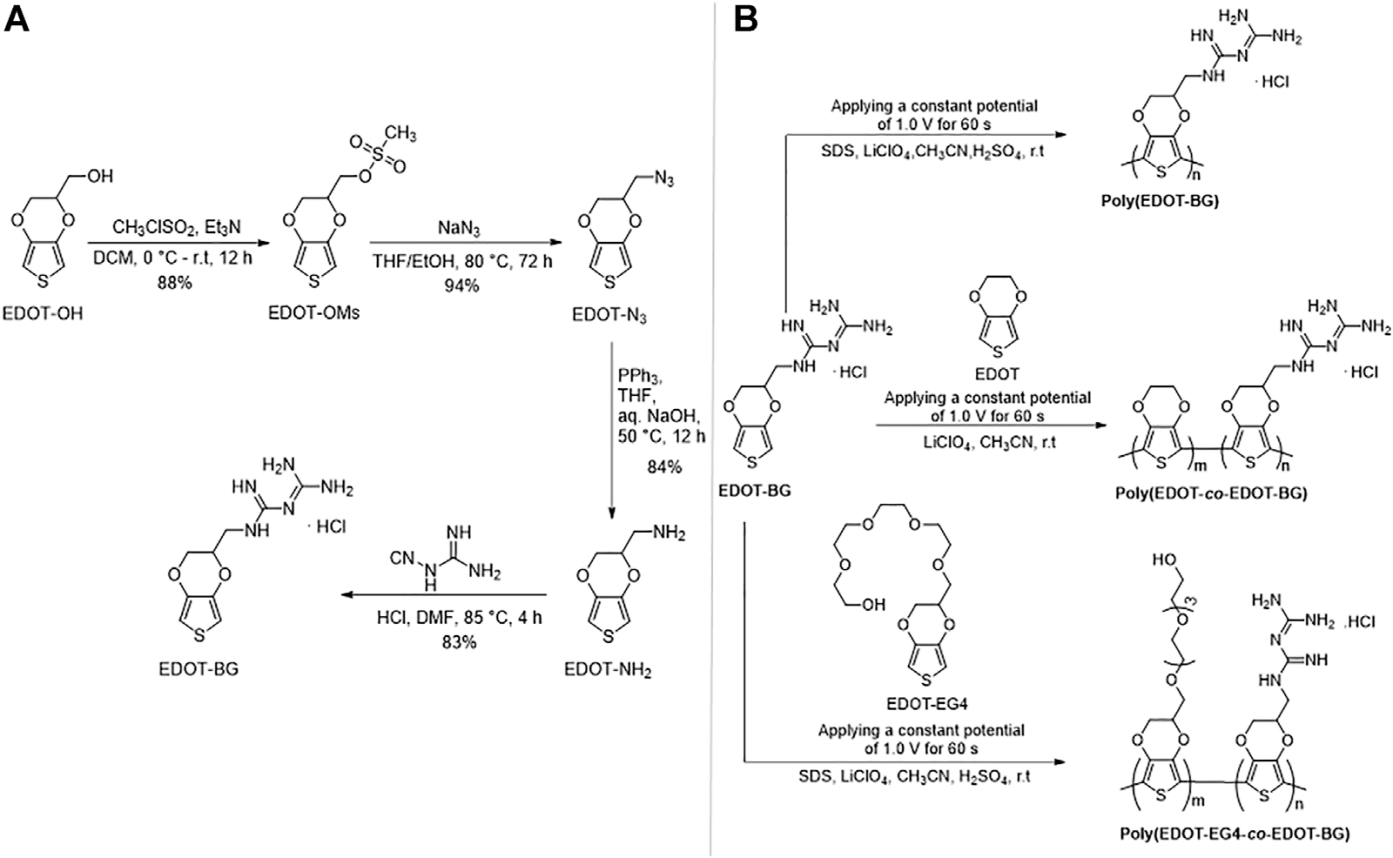
**(A)** Synthesis route of the biguanide hydrochloride-functionalized 3,4-ethyledioxythihiophene (EDOT-BG) monomer and **(B)** electrodeposition of poly(EDOT-BG), poly(EDOT-*co*-EDOT-BG), and poly(EDOT-EG4-*co*-EDOT-BG) copolymers on ITO-glass slides.

Primarily, amino-functionalized EDOT (EDOT-NH_2_) was synthesized from the commercially available hydroxymethyl EDOT (EDOT-OH) starting material in a few steps. Briefly, EDOT-OH was first mesylated to give the EDOT-OMs intermediate. The EDOT-OMs were reacted with sodium azide to give azide-functionalized EDOT (EDOT-N_3_) via nucleophilic substitution. EDOT-N_3_ was then reduced to give EDOT-NH_2_. Finally, EDOT-NH_2_ was reacted with dicyandiamide in aq. HCl to give the biguanide hydrochloride-functionalized EDOT (EDOT-BG) product in a moderately high yield (Scheme 1a). The detailed synthesis procedure for EDOT-NH_2_ is presented in the Supplementary Information. The structure of EDOT-BG was characterized by ^1^H and ^13^C NMR spectroscopy and mass spectrometry techniques (Supplementary Information). In addition, tetra ethylene glycol-functionalized EDOT (EDOT-EG4) was synthesized following the reported procedure (Luo et al., 2012).

The polymers were prepared through the electropolymerization method, which is a straightforward technique to deposit polymers directly onto a conducting substrate. Hence, the biguanide-functionalized PEDOT homopolymers and copolymers were electropolymerized onto ITO-glass slides successfully after a series of optimizations. However, initially, the electrodeposition of poly(EDOT-BG) was difficult due to less solubility of the EDOT-BG monomer in commonly used organic solvents for electropolymerization such as MeCN and CH_2_Cl_2_. On the other hand, electropolymerization in an aqueous solvent resulted in less stable films that dissolve easily and could not attach on the ITO-glass slides. In addition, the electropolymerization in a mixture of H_2_O and MeCN was also unsuccessful due to the dissolution of the film in this solvent system. Further electropolymerization efforts using ionic liquids such as 1-butyl-3-methylimidazolium hexafluorophosphate (BMIMPF_6_) and 1-butyl-3-methylimidazolium octyl sulfate (BMIM OSU) as both a supporting electrolyte and solvent were also unsuccessful.

Previously, we faced such difficulties during the electropolymerization of zwitterionic phosphorylcholine bearing EDOT (EDOT-PC) due to the insolubility of the monomer in MeCN or other common solvents, which was later circumvented by the addition of a surfactant. The addition of the surfactant enhanced the EDOT-PC solubility in MeCN, which led to successful electrodeposition of poly(EDOT-PC) (Zhu B. et al., 2014). Similarly, here, the sodium dodecyl sulfate (SDS) surfactant (20 mM) was added to the EDOT-BG suspension in MeCN forming a turbid solution which was further dissolved by the addition of conc. H_2_SO_4_ (3%, v/v), which resulted in a clear monomer solution. Thus, a thin purple poly(EDOT-BG) polymer film was electrodeposited on ITO-glass successfully when a constant potential of 1.0 V was applied for 60 s in the presence of LiClO_4_ (0.1 M) supporting electrolyte. The electrodeposition was performed with a three-electrode system electrochemical cell set-up using ITO-glass as a working electrode, Ag/AgNO_3_ as a reference electrode, and Pt wire as a counter electrode (Figure 1A).

The property of polymers can be tuned through copolymerization of different monomers each with a specific function. To examine the effect of biguanide concentration on antimicrobial properties, EDOT-BG was copolymerized with unfunctionalized EDOT from a mixture of equimolar monomers. A dark-blue poly(EDOT-*co*-EDOT-BG) copolymer film was formed as confirmed by structural analysis data. Previously, we have demonstrated the antifouling properties of oligoethylene glycol-functionalized PEDOT polymers that resisted protein adsorption and cell adhesion on various surfaces (Zhao et al., 2013). Consequently, herein, we copolymerized EDOT-BG with tetra (ethylene glycol)-functionalized EDOT (EDOT-EG4) to create multifunctional (electroactive, antimicrobial, and antifouling) surface coatings. Hence, the equimolar feed ratio of EDOT-EG4 and EDOT-BG was mixed to give a final concentration of 10 mM and electropolymerized on ITO-glass slides to give a thin light purple poly(EDOT-EG4-*co*-EDOT-BG) copolymer. PEDOT was also electrodeposited on ITO-glass slides and used as a reference material for the antimicrobial and antifouling studies on biguanide-functionalized PEDOTs (Figure 1B; Scheme 1B).

### 3.2 Characterizations of the Polymer Films

#### 3.2.1 UV/Vis Spectroscopy

**FIGURE 2:**
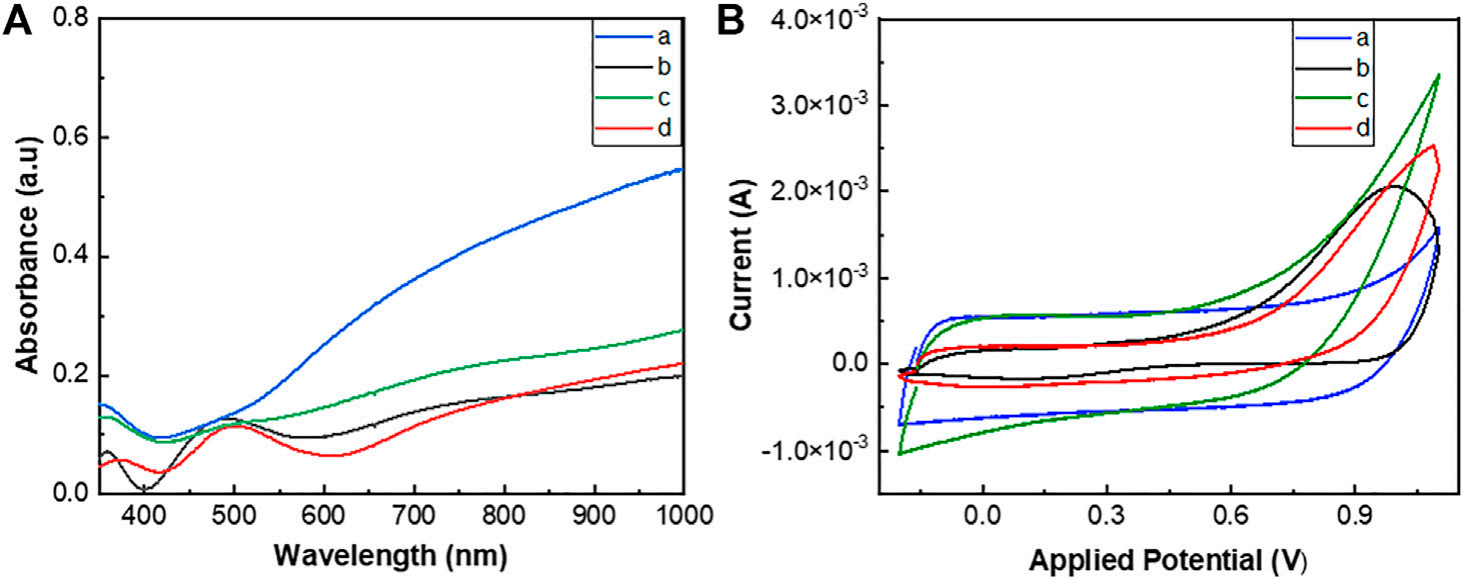
UV/Vis spectra **(A)** and cyclic voltammograms **(B)** of PEDOT (a), poly(EDOTBG) (b), poly(EDOT-*co*-EDOT-BG) (c), and poly(EDOT-EG4-*co*-EDOT-BG) (d) films electrodeposited on ITO-glass slides. The cyclic voltammogram was recorded using Ag/AgCl as a reference electrode and Pt wire as a counter electrode in PBS with 10 mM [Fe(CN)]^3-/4-^ redox couple at a scan rate of 100 mV/s.

The electrodeposition of the polymers on ITO-glasses was confirmed by UV/Vis spectral analysis of the films. PEDOT and poly(EDOT-*co*-EDOT-BG) showed blue-colored thicker films, while poly(EDOT-BG) and poly(EDOT-EG4-*co*-EDOT-BG) showed light purple thinner films (Figure 2A). Generally, the UV/Vis spectra of the polymers showed two broad absorption peaks at around 500 nm and 700–800 nm due to the π → π* transition of the thiophene ring and polarons, respectively, which is a common property of doped PEDOT derivatives.

#### 3.2.2 Energy-Dispersive X-Ray and X-Ray Photoelectron Spectroscopy Measurements

**FIGURE 3:**
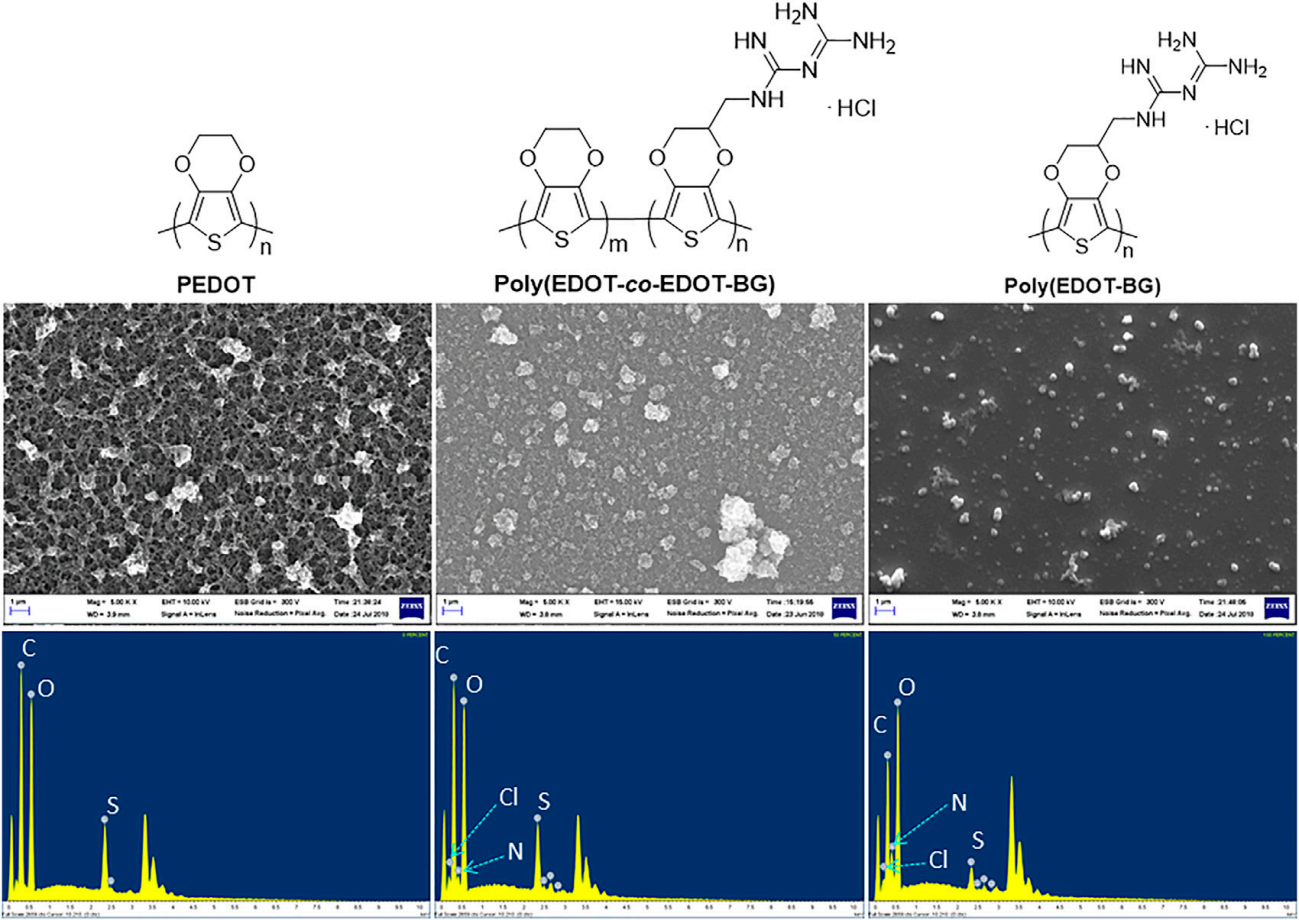
SEM images (upper) and EDX elemental mapping (lower) of PEDOT, poly(EDOT-*co*-EDOT-BG), and poly(EDOT-BG) polymers electrodeposited on ITO-glass slides.

In addition, EDX elemental mapping of the films also confirmed the successful electrodeposition of PEDOT and poly(EDOT-BG) homopolymers and the poly(EDOT-*co*-EDOT-BG) copolymer. The detailed quantitative analyses of the atomic elements from the EDX spectra show distinct signals from N and Cl atoms for poly(EDOT-BG) and poly(EDOT-*co*-EDOT-BG) films, which are the constituent elements of the biguanide hydrochloride functional group (Figure 3). In addition, the polymer films produced signals from C, O, and S, which are the constituent elements of EDOT as indicated in Table 1.

**TABLE 1:** Atomic elements of PEDOT, poly(EDOT-*co*-EDOT-BG), and poly(EDOT-BG) polymer films electrodeposited on ITO-glass slides.

Element	PEDOT		PEDOT-*co*-EDOT-BG		PEDOT-BG	
	*Wt*%	*At*%	*Wt*%	*At*%	*Wt*%	*At*%
C K	48.53	58.38	44.51	53.88	29.58	36.00
N K	-	-	6.93	7.19	17.17	17.92
O K	40.72	36.77	37.30	33.90	47.74	43.61
S K	10.75	4.85	9.42	4.27	4.49	2.05
Cl K	-	-	1.84	0.75	1.02	0.42

**FIGURE 4:**
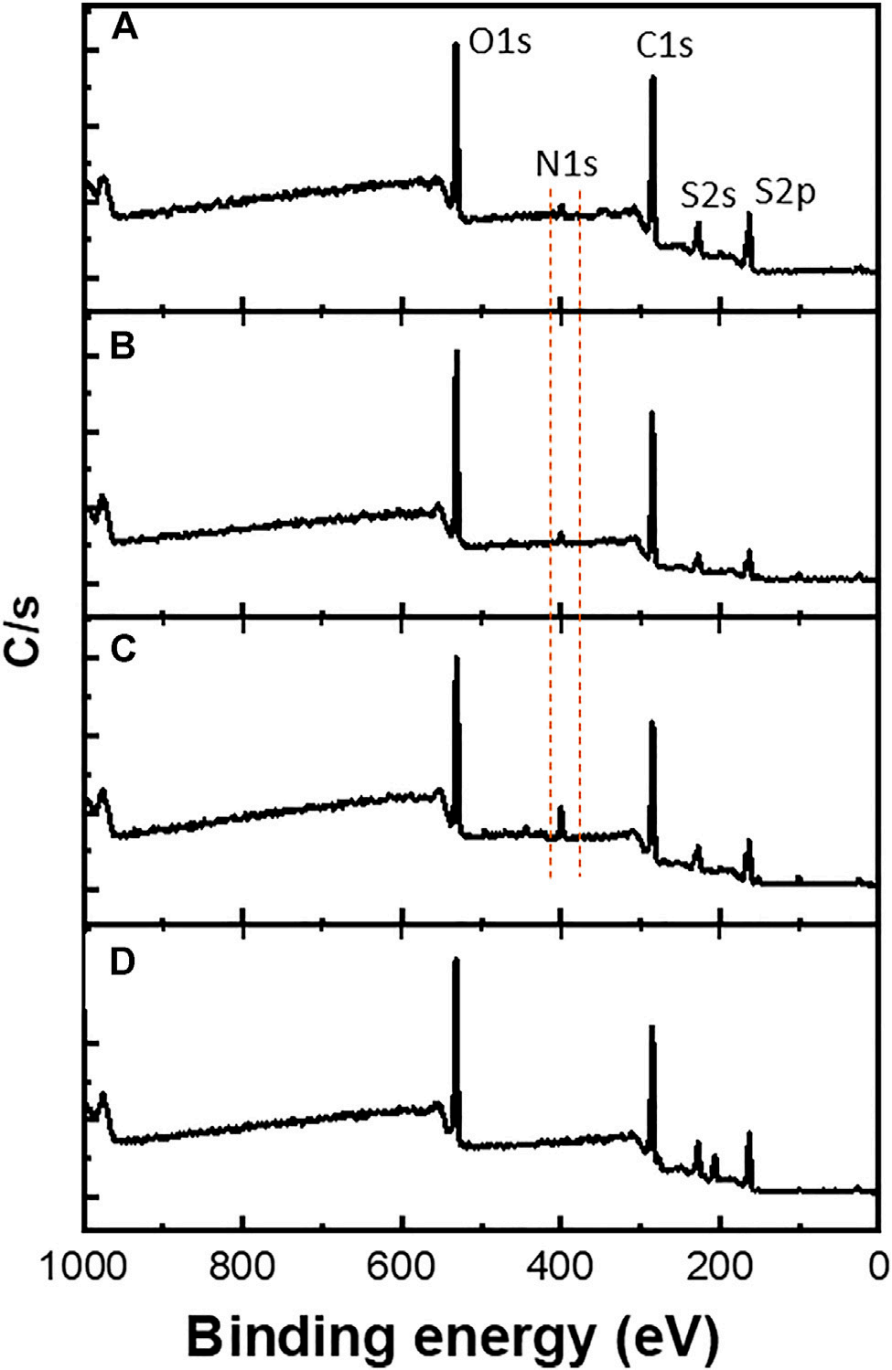
XPS spectra of polymer films electrodeposited on ITO-glass slides. The dotted line indicates the characteristic N atom peak observed from poly(EDOT-BG) **(C)**, poly(EDOT-*co*-EDOT-BG) **(B)**, and poly(EDOT-EG4-*co*-EDOT-BG) **(A)** films that confirmed the successful copolymerization. **(D)** represents PEDOT spectrum as a reference.

Furthermore, XPS measurements were performed mainly to confirm the copolymerization of EDOT-BG with either EDOT or EDOT-EG4. As shown in Figure 4, a characteristic peak for the nitrogen element (N 1s peak) was observed at ∼400 eV from the poly(EDOT-BG) homopolymer and copolymers (poly(EDOT-*co*-EDOT-BG) and poly(EDOT-EG4-*co*-EDOT-BG)). XPS spectra are in agreement with a previous report (Zhi et al., 2017). As expected, the N peak intensity of the copolymers is lower compared to the poly(EDOT-BG) homopolymer, supporting the EDX elemental mapping.

#### 3.2.3 Scanning Electron Microscopy Images of Polymer Films

Field emission scanning electron microscopy (FE-SEM) was utilized to study the morphology of the electrodeposited films. The SEM micro image of the PEDOT film indicated its characteristic nano-wire-like morphology, while poly(EDOT-*co*-EDOT-BG) and poly(EDOT-BG) films showed granular and smooth morphologies, respectively (Figure 3).

### 3.3 Electrochemical Properties of the Polymers

The electroactivity of electrically conducting polymers is an important factor in the use of such materials in the manufacturing of various devices such as bioelectronics. Hence, the electrochemical properties of the electrodeposited polymers were analyzed by the CV technique in PBS buffer using 10 mM [Fe(CN)6]^3-/4-^ as the redox couple. The cyclic voltammogram was recorded using a three-electrode system autolab potentiostat using Ag/AgCl as a reference electrode, Pt wire as a counter electrode, and polymer-coated ITO-glass slides as the working electrode. The cyclic potential for each polymer was scanned between −0.2 and 0.6 V at a scan rate of 100 mV/s over two cycles. The voltammograms of the respective polymer films showed the corresponding oxidation/reduction peaks associated with the mass transfer of [Fe(CN)6]^3-/4-^ and polymers that confirmed the electroactivity of the polymers. As a result, oxidation peak potentials of 0.35, 0.53, 0.46, and 0.43 V as well as reduction peak potentials of 0.07, −0.06, −0.024, and 0.022 V were obtained for PEDOT, poly(EDOT-BG), poly(EDOT-*co*-EDOT-BG), and poly(EDOT-EG4-*co*-EDOT-BG) polymers, respectively (Figure 2B). Surprisingly, poly(EDOT-*co*-EDOT-BG) and poly(EDOT-EG4-*co*-EDOT-BG) showed intermediate oxidation/reduction peak potential values compared to PEDOT and poly(EDOT-BG) homopolymers, which is another confirmation for successful electrodeposition of copolymers in agreement with XPS and EDX results.

### 3.4 Cell Viability (Biocompatibility) Studies

**FIGURE 5:**
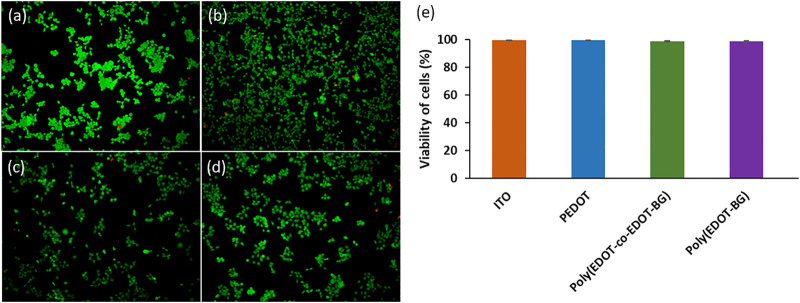
Examples of fluorescence microscopy images of a live/dead cell assay of HEK cells cultured for 48 h on **(A)** bare ITO-glass slide, **(B)** PEDOT, **(C)** poly(EDOT-*co*-EDOT-BG), and **(D)** poly(EDOT-BG) polymer films; **(E)** quantitative cell viability graph (%).

The biocompatibility of PEDOT-based materials has been reported by various researchers (Luo et al., 2008). Similarly, herein, we investigated the biocompatibility of the biguanide-functionalized PEDOT derivatives from cell viability studies using a human embryonic kidney 293T (HEK-293T) cell as a model. A bare ITO-glass slide was used as a reference. The HEK-293T cells were seeded on each blank and polymer-coated ITO-glass slides at a concentration of 2 × 10^4^ cells/cm^2^ and incubated for 48 h at 37°C in the presence of 5% CO_2_. The viability of HEK-293T cells was then determined from fluorescence microscopy images of live (green) and dead (red) cells stained with a live/dead cell viability assay kit (Figures 5A–D). The quantitative analysis was carried out by counting the live and dead cells, and the results confirmed the biocompatibility of poly(EDOT-BG) and poly(EDOT-*co*-EDOT-BG) polymers with above 98.5% cell viability, nearly the same value compared with a bare-ITO glass slide and PEDOT polymer (∼99% viability) (Figure 5E). This confirmed that the biguanide-functionalized PEDOT polymer films are less toxic and can be used as coating materials for domestic and biomedical devices including implantable bioelectronics. In addition to the live/dead cell viability assay, the biocompatibility of the polymers was determined from the MTT viability assay. Similarly, the MTT assay also confirmed the biocompatibility of the biguanide-functionalized PEDOTs with comparable cell viability of poly(EDOT-BG) (95.7%) to unfunctionalized PEDOT (96.1%) (Supplementary Figure S2).

### 3.5 Evaluation of Antimicrobial Activities

The antimicrobial properties of the biguanide-functionalized PEDOT derivatives were examined on *E. coli* as a model bacterial strain. Bare ITO-glass and unfunctionalized PEDOT were used as references. All samples (bare ITO-glass, PEDOT, poly(EDOT-BG), and poly(EDOT-*co*-EDOT-BG)) were cut into 1 × 1 cm size, sterilized, and placed in a 12-well cell culture plate. 50 µL of *E. coli* (10^6^ cells/mL) in PBS was spread on each sample and incubated for 2 h at 37°C. After the incubation, the samples were washed with PBS, and antibacterial activities were determined from the OD measurement at 600 nm by a plating method and live/dead staining assay.

**FIGURE 6:**
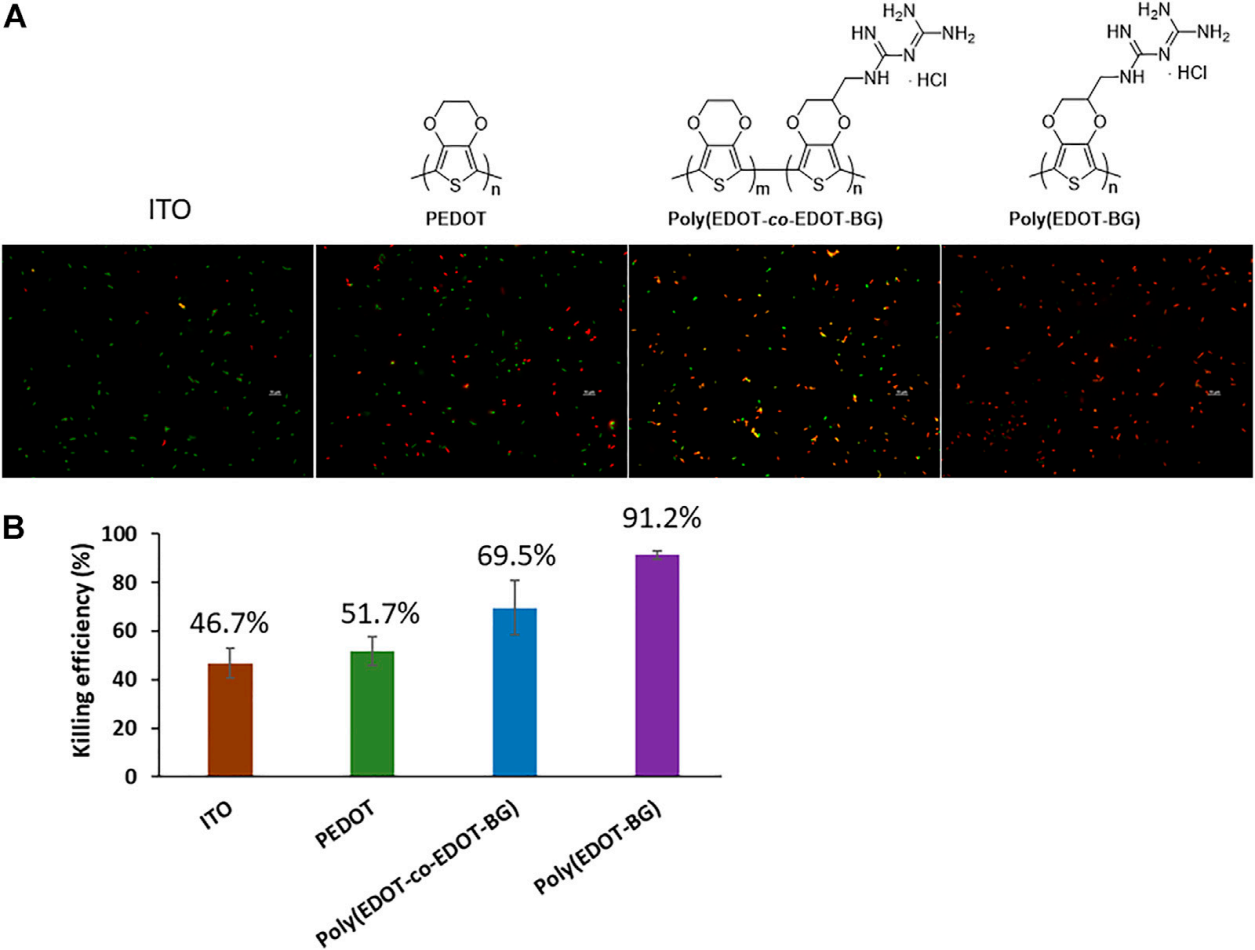
Bactericidal efficiency of the polymers on *E. coli*. **(A)** Fluorescence microscopy images of live (green) and dead (red) bacteria cells cultured on different substrates (incubated for 2 h at 37°C) and **(B)** graph showing the bactericidal efficiency (%).

The OD was recorded at 600 nm for the suspension culture obtained after the incubation and plated on a Luria broth (LB) agar plate. The OD measurement for the suspension of each sample indicated a decreasing value in the order of ITO-glass > PEDOT > poly(EDOT-*co*-EDOT-BG) > poly(EDOT-BG). Similarly, a higher number of bacterial colonies were observed on bare ITO-glass slides. In contrast, very few colonies could be formed on poly(EDOT-BG) films, which demonstrated the efficient bactericidal activity of biguanide-functionalized PEDOTs (Supplementary Figure S1). Further bactericidal activity was examined from fluorescence microscopy images of the bacteria on the surface of each polymer film (after 2 h incubation) stained with a live/dead bacterial assay kit (Invitrogen, Thermo Fisher, United States) for 15 min in the dark. The bactericidal efficiency was thus determined by counting the live (green) and dead (red) bacteria cells. As shown in Figure 6, the poly(EDOT-BG) homopolymer showed a higher bacterial killing efficiency above 91%, while PEDOT and poly(EDOT-*co*-EDOT-BG) showed killing efficiencies of 52 and 70%, respectively. It is noteworthy that the composition of the biguanide moiety is critical to the bactericidal activity as observed from the killing efficiency of the poly(EDOT-BG) homopolymer and poly(EDOT-*co*-EDOT-BG) copolymer. Although the detailed killing mechanism from biguanide-functionalized PEDOTs is not studied here, we believe that it might be due to a strong interaction between the cationic biguanide moiety and bacterial cell membrane components to disrupt the membrane and hence release the contents as mentioned elsewhere (Bottcher et al., 2013; Chindera et al., 2016; Zhi et al., 2017). The biguanide group in our material is covalently immobilized on the substrate; hence, the bacterial killing mechanism would be different from other similar biguanide moiety-containing molecules that can penetrate the cell membrane and cause chromosome condensation (Chindera et al., 2016).

### 3.6 Cell Adhesion and Protein Adsorption Properties of the Poly(EDOT-EG4-*co*-EDOT-BG) Copolymer

The antifouling nature of a material is vital for prolonged use of the device, especially for implantable biomedical devices. Protein molecules from the extracellular matrix may first bind to the surface of the material, making the surface prone to cell adhesion. The cells will then bind to the surface and hinder the function of the material after long exposure. To tackle such problems, materials with antifouling properties are desirable, which can protect protein adsorption and hence cell adhesion. Poly(ethylene glycol)-functionalized polymers are one of the best candidates in the preparation of such antifouling surfaces (Zhao et al., 2013). Hence, tetra ethylene glycol-functionalized EDOT (EDOT-EG4) was electrochemically copolymerized with EDOT-BG to create the multifunctional poly(EDOT-EG4-*co*-EDOT-BG) copolymer with simultaneous antimicrobial and antifouling properties.

**FIGURE 7:**
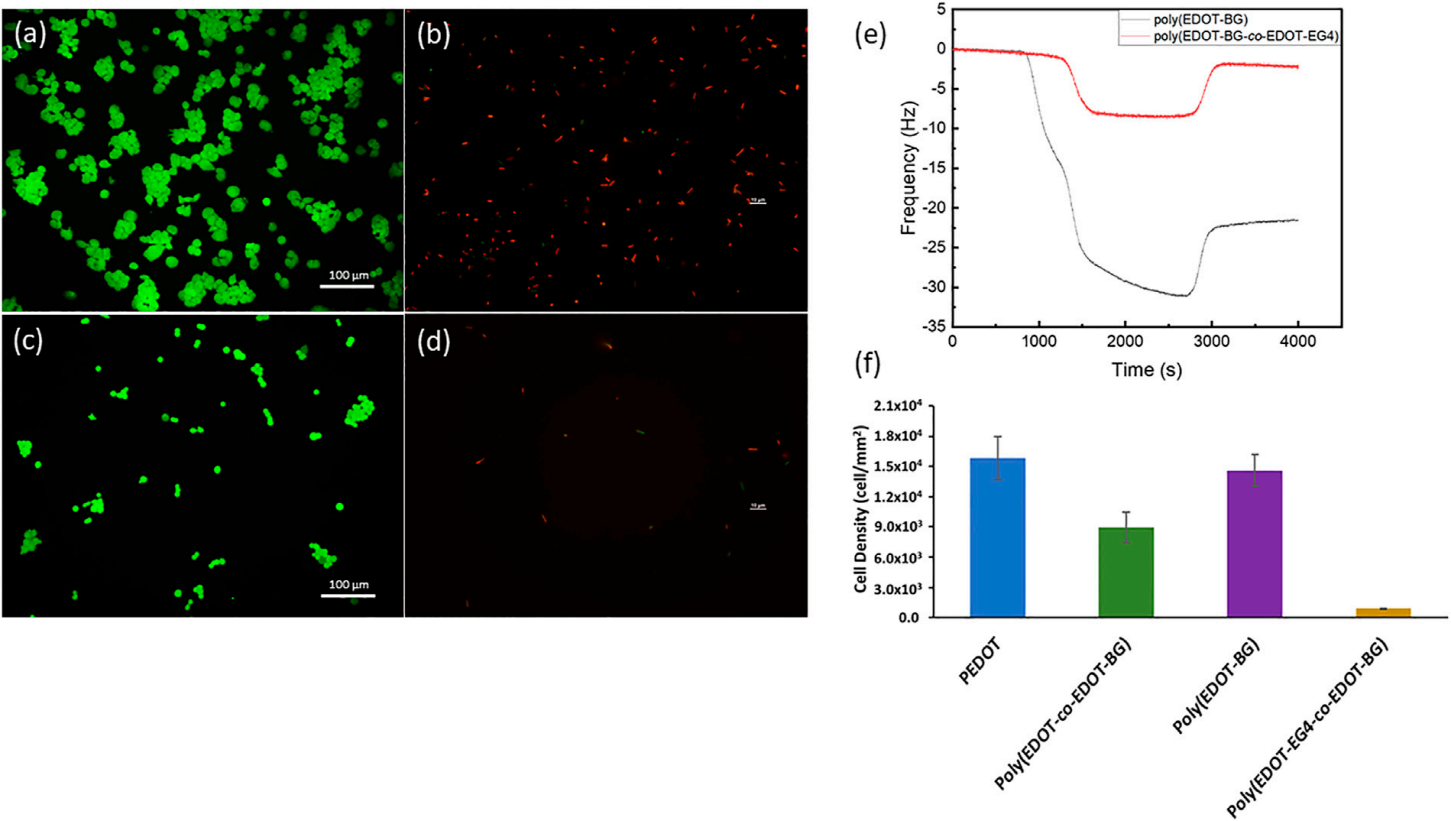
Antifouling properties of the poly(EDOT-EG_4_-*co*-EDOT-BG) polymer from cell adhesion and protein adsorption studies. Representative fluorescence microscopy image of HEK 293T cells (stained with green staining dye) attached on poly(EDOT-BG) **(A)** and poly(EDOT-EG_4_-*co*-EDOT-BG) polymers **(C)**; fluorescence microscopy image of *E. coli* (stained with red staining dye) attached on poly(EDOT-BG) **(B)** and poly(EDOT-EG_4_-*co*-EDOT-BG) **(D)**. QCM measurements for protein adsorption on poly(EDOT-BG) and poly(EDOT-EG_4_-*co*-EDOT-BG) films electrodeposited on a gold QCM crystal **(E)** and quantitative cell density of HEK 293T cells cultured for 48 h on PEDOT, poly(EDOT-BG), poly(EDOT-*co*-EDOT-BG), and poly(EDOT-EG_4_-*co*-EDOT-BG) polymers electrodeposited on ITO-glass slides **(F)**. The scale bar for Figures **(B)** and **(D)** is 10 µm.

First, the antifouling property was evaluated from protein binding studies. The protein binding property was monitored on QCM using bovine serum albumin (BSA) (1 mg/ml) as a model protein. The poly(EDOT-BG) homopolymer was used as a reference. Both poly(EDOT-BG) and poly(EDOT-EG4-*co*-EDOT-BG) films were electropolymerized on gold QCM sensor crystals through the potentiostatic technique. The *in situ* protein binding was thus assessed from the difference in change of frequency (Δf) between poly(EDOT-BG) and poly(EDOT-EG4-*co*-EDOT-BG) due to protein adsorption on the surfaces. As can be seen from Figure 7E, the decrease in frequency was more pronounced for poly(EDOT-BG) compared to poly(EDOT-EG4-*co*-EDOT-BG) during the passage of BSA solution over the QCM crystal. After saturation, the fluent was switched to PBS buffer solution to remove loosely bound protein from the surfaces. Only a small rise in the frequency of poly(EDOT-BG)-coated crystals was observed compared to the frequency of the poly(EDOT-EG4-*co*-EDOT-BG) copolymer, confirming a larger amount of protein adsorption on the former. The poly(EDOT-BG) polymer showed a higher Δf (22 Hz) compared to Δf (∼1 Hz) for poly(EDOT-EG4-*co*-EDOT-BG), confirming the excellent antifouling nature of the poly(EDOT-EG4-*co*-EDOT-BG) copolymer. Furthermore, the antifouling property of poly(EDOT-EG4-*co*-EDOT-BG) was determined from a cell adhesion study using HEK-293T cells by comparing the cell densities obtained after 48 h with respect to PEDOT, poly(EDOT-BG), and poly(EDOT-*co*-EDOT-BG) polymers. As shown in Figures 7A,C,F, very few cells could attach on poly(EDOT-EG4-*co*-EDOT-BG) compared to other polymers in agreement with previous studies of oligo ethylene glycol-functionalized materials (Wu et al., 2019).

Finally, both the antifouling and antimicrobial properties of poly(EDOT-EG4-*co*-EDOT-BG) were studied using *E. coli* as model bacteria. A very small amount of bacterial cells could attach on poly(EDOT-EG4-*co*-EDOT-BG) compared to those attached on the poly(EDOT-BG) homopolymer. Furthermore, those small numbers of attached *E. coli* on poly(EDOT-EG4-*co*-EDOT-BG) are dead cells as can be seen from the fluorescence image (Figures 7B,D, F). This confirmed that the copolymerization helped to integrate the antifouling property of EDOT-EG4 with the antimicrobial nature of the poly(EDOT-BG) surface without affecting the bactericidal activities much.

## 4 Conclusion

In summary, novel biguanide-functionalized PEDOT derivatives, poly(EDOT-BG), poly(EDOT-*co*-EDOT-BG), and poly(EDOT-EG4-*co*-EDOT-BG) polymers, were designed and prepared through electropolymerization on different substrates. The bactericidal activity studies on *E. coli* demonstrated the antimicrobial properties of the polymers with bacterial killing efficiencies of 70 and 91% for poly(EDOT-*co*-EDOT-BG) and poly(EDOT-BG) polymers, respectively. The lower bactericidal efficiency of poly(EDOT-*co*-EDOT-BG) compared to the poly(EDOT-BG) homopolymer confirmed the importance of biguanide concentration on the antimicrobial activity. Further copolymerization of EDOT-BG with EDOT-EG4 resulted in a dual-purpose poly(EDOT-EG4-*co*-EDOT-BG) copolymer with simultaneous antimicrobial and antifouling properties. The poly(EDOT-EG4-*co*-EDOT-BG) copolymer resists both protein adsorption and mammalian/bacterial cell adhesions in addition to killing those small numbers of attached bacteria on the surface. With their less toxicity to mammalian cells, the biguanide-functionalized PEDOT derivatives can have a potential application in the preparation of multifunctional electroactive surface coatings for domestic touch-screen and bioelectronic devices.

## Data Availability

The original contributions presented in the study are included in the article/Supplementary Material; further inquiries can be directed to the corresponding author.
