# Supplementary material for: Biguanide- and Oligo(Ethylene Glycol)-Functionalized Poly(3,4-Ethylenedioxythiophene): Electroactive, Antimicrobial, and Antifouling Surface Coatings
Source: Front Chem. 2022 Aug 5;10:955260. doi: 10.3389/fchem.2022.955260 (PMC9389217; doi:10.3389/fchem.2022.955260)
Supplement: Supplementary file 1 [file DataSheet1.pdf]

Supplementary Material

Chemicals

Hydroxymethyl EDOT (EDOT-OH) (Angene); 3,4-ethylenedioxythiophene (EDOT), sodium azide and KBr (Sigma Aldrich); triethylamine (Et_3N), methane sulfonyl chloride, triphenylphosphine and dicyandiamide (Alfa Aesar); $MgSO_4$ and sodium dodecyl sulfate (SDS) (J. T. Baker) and all other chemicals used as received without further purification. Tetra ethylene glycol functionalized-EDOT (EDOT-EG_4) was prepared following reported procedure (Chen and Luo, 2015). The reactions were performed using dry solvents under a nitrogen atmosphere.

Synthesis of EDOT-NH_2

EDOT-NH_2 was synthesized in three steps from (2,3-Dihydrothieno[3,4-b][1,4]dioxin-2-yl)methanol (EDOT-OH) starting material as follows.

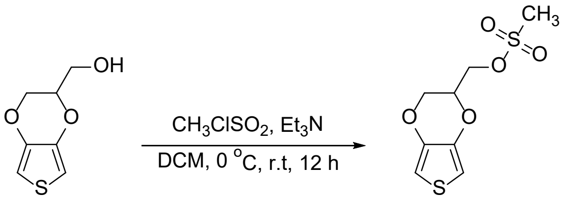

Synthesis of (2,3-dihydrothieno[3,4-b][1,4]dioxin-2-yl)methyl methanesulfonate (EDOT-O-SO_2CH_3). EDOT-OH (2.0 g, 11.63 mmol) was added to a 100 mL two-neck round-bottom flask with a stir bar and backfilled with N_2. Anhydrous CH_2Cl_2 (20 mL) and Et_3N (1.94 mL, 1.41 g, 14.0 mmol) were added via syringe and cooled to 0 °C. Methanesulfonyl chloride (1.08 mL, 1.6 g, 14 mmol) was added dropwise and stirred overnight at room temperature. Water was added to the reaction mixture and the aqueous layer was extracted with CH_2Cl_2 (2x). The combined organic layers were washed with 5% H_2SO_4, saturated $NaHCO_3$ and brine, dried over $MgSO_4$ and concentrated. The crude product was purified using silica gel column in hexane with gradual increase of ethyl acetate from 10 to 50% to yield white solid when kept in fridge (2.56 g, 88%). ^{1}H NMR (400 MHz, $CDCl_3$) δ: 6.36 (s, 2H), 4.43 – 4.40 (m, 3H), 4.24 (d, 1H, J = 11.8), 4.09 (dd, 1H, J = 11.8, 5.9 Hz), 3.06 (s, 3H). ^{13}C NMR (100 MHz, $CDCl_3$) δ: 141.1, 140.6, 100.6, 100.58, 71.3, 67.0, 65.1, 37.9.

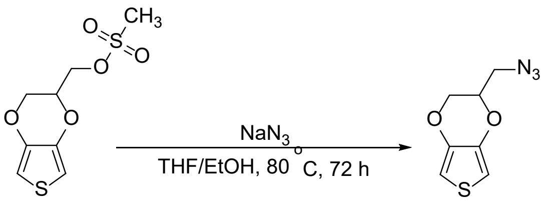

Synthesis of 2-(azidomethyl)-2,3-dihydrothieno[3,4-b][1,4]dioxine (EDOT-N_3). EDOT-O-SO_2CH_3 (1.6 g, 6.4 mmol) was added to a 100 mL two-neck round-bottom flask and backfilled with N_2. THF (6 mL) and EtOH (10 mL) were added via syringe followed by addition of freshly prepared aqueous NaN_3 solution (4.16 g, 64 mmol in 15 mL water). The reaction mixture was stirred at 80 °C for 72 h. The majority of THF was removed by a rotary evaporator, and the aqueous layer was extracted with

ethyl acetate (3x), dried ($MgSO_4$) and concentrated under a rotary evaporator. The crude product was purified with silica gel column (hexane:ethyl acetate; 9.5:0.5, v/v) to yield colorless oil (1.18 g, 94%). ^{1}H NMR (400 MHz, $CDCl_3$) δ: 6.36 (d, 1H, J = 4.0 Hz), 6.34 (d, 1H, J = 3.5 Hz), 4.32 – 4.27 (m, 1H), 4.17 (dd, 1H, J = 11.6, 1.9 Hz), 4.03 (dd, 1H, J = 11.7, 6.8 Hz), 3.55 (dd, 1H, J = 13.1, 6.1 Hz), 3.47 (dd, 1H, J = 13.2, 5.2 Hz). ^{13}C NMR (100 MHz, $CDCl_3$) δ: 141.3, 140.9, 100.5, 100.3, 72.6, 66.0, 50.7.

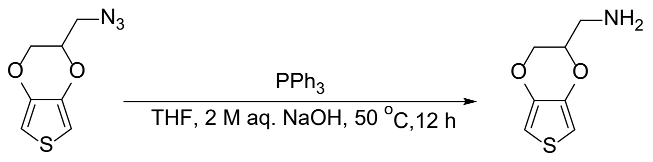

Synthesis of (2,3-dihydrothieno[3,4-b][1,4]dioxin-2-yl)methanamine (EDOT-NH₂). EDOT-N_3 (1.5 g, 7.61 mmol) and PPh_3 (2.19 g, 8.38 mmol) were dissolved in THF (10 mL) in a 100 mL two-neck round-bottom flask and stirred at 50 °C under N_2 atmosphere. Aqueous NaOH (2M, 10 mL) was added dropwise using syringe and the reaction mixture was allowed to stir overnight. The reaction mixture cooled to room temperature and the majority of THF was removed by rotary evaporator. 2M HCl solution was added to lower the pH below 3 and extracted with CH_2Cl_2 (3x). The organic layer was discarded and 1 M aqueous NaOH solution was added to adjust the pH of the aqueous layer above 10. The aqueous layer was then extracted with CH_2Cl_2 and the organic layers were dried ($MgSO_4$) and concentrated to yield viscous oil (1.1 g, 84%). ^{1}H NMR (400 MHz, $CDCl_3$) δ: 6.30 (d, 2H, J = 3.4 Hz), 4.19 – 4.12 (m, 1H), 4.08 (dd, 1H, J = 10.7, 6.2 Hz), 3.97 (dd, 1H, J = 11.2, 7.8 Hz), 2.99 – 2.89 (m, 2H), 1.36 (b, s, 2H). ^{13}C NMR (100 MHz, $CDCl_3$) δ: 141.9, 141.8, 99.7 (2C), 75.4, 66.8, 42.6.

Bacterial sample preparation and antimicrobial property evaluations

Microorganisms and Growth Conditions

The antibacterial and antifouling study was performed using gram-negative Escherichia coli (E. coli). The bacteria were grown at 37 °C for 16 h at 180 rpm in nutrient broth (LB broth) (Difco, Detroit, MI) prepared in DI water. A 100 µL culture was taken from this and then added to 5 mL of fresh nutrient broth which was grown for 6 h at 37 °C and 180 rpm. From this culture, 1 mL culture was centrifuged at 10,000 rpm for 5 min. The obtained pellet was washed twice with PBS to remove excess medium and resuspended the pellet in PBS. 50 µL of 1 x 106 CFU/mL of bacterial concentration was used for further experiments using colony counting OD method.

Evaluation of Bactericidal Efficiency

To evaluate antibacterial activity, the bacterial culture was incubated with the samples on orbital shaker at 80 rpm /2 hours at 37oC. After the incubation, the samples were washed with PBS and antibacterial activities for all the samples were analyzed by recording the OD (600nm), plating method and Live/Dead Staining Assay.

Plating and OD (Optical Density) method

The microbial suspension treated with the samples was collected, and diluted in PBS, spread onto nutrient agar (LB Agar), and incubated at 37 °C for 18 h. 200 µL of suspension collected from the surface was used to record OD at 600nm for each sample. Bactericidal activities of the samples were determined by comparing colony counts (qualitatively) and OD between different samples.

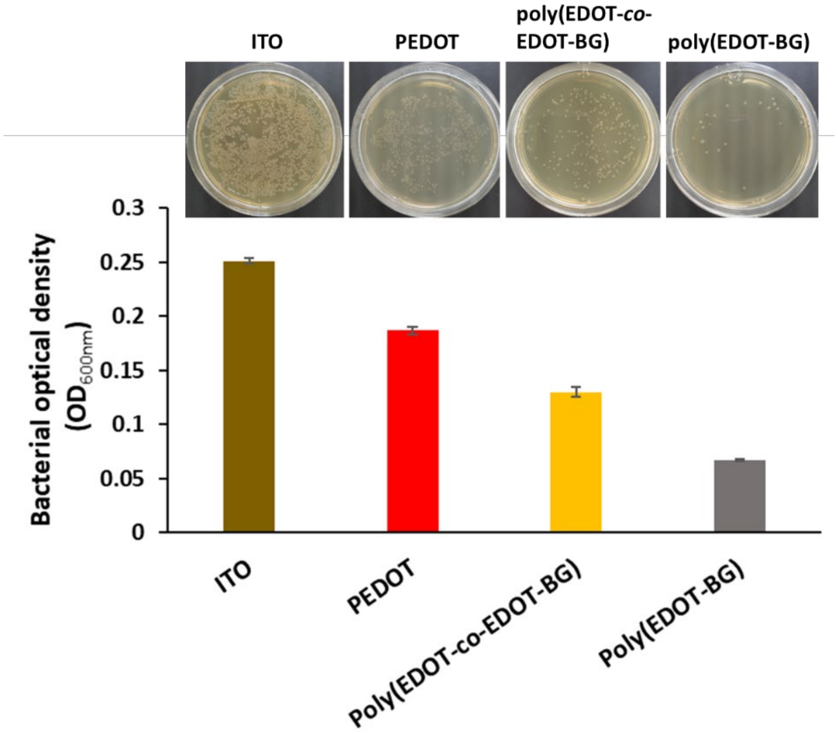

Figure S1. Photograph showing bacterial colonies formed on LB agar plate after 18 h incubation spread from bacterial suspension treated with the polymer samples for 2 h (upper), and a graph showing OD_{600} measurements of bacterial suspension collected after 2 h incubation on each polymer coated sample.

Cytotoxicity Studies

Live/Dead Staining Assay

A live/dead staining assay was performed to evaluate the killing efficiency of the surfaces. The surfaces were incubated with E. coli bacteria (1 × 106 cells/mL in phosphate buffered saline (PBS), pH 7.4) for 2 hours at 37 °C, and then the surface was washed with sterile PBS. The surfaces were stained with a Live and dead bacterial assay kit (Thermofisher Scientific, Cat. L7007, a green-fluorescent nucleic acid stain which generally labels all bacteria and red-fluorescent nucleic acid stain which penetrates only bacteria with damaged membranes) for 15 min in the dark. After gently rinsing with sterile water and drying in air, the surface-attached bacteria were examined using a fluorescence microscope (IX71, Olympus, Japan) with a 40X objective lens. The 3 representative images were chosen for each surface. Three replicates were performed and the relative number of live (green) vs dead (red or yellow) bacteria were counted and the results are presented in mean ± SD.

MTT assay

In addition to live/dead cell viability assay studies, the cytotoxicity of biguanide containing PEDOTs (poly(EDOT-co-EDOT-BG) and poly(EDOT-BG)) were evaluated using the MTT (3-(4,5-dimethylthiazol-2-yl)-2,5-diphenyltetrazolium bromide) assay. Similar to live/dead cell assay method, HEK-293T cell lines were used for MTT assay studies. Bare ITO-glass and PEDOT were used as

references. The cells were grown for 24 hours in DMEM culture media and the cytotoxicity study was evaluated according to the following procedure.

A 5 mg/mL MTT reagent was prepared and stored at 4 °C in dark until use. The stock solution of MTT was diluted 10-fold in the culture medium. To evaluate the in vitro cytotoxicity, the cell lines were seeded at a density of 1 × 104 cells/mL on 1 x 1 cm samples (bare ITO-glass, PEDOT, poly(EDOT-co-EDOT-BG), and poly(EDOT-BG)) placed in 12-well cell culture plates and incubated for 24 hours. After incubation, the culture media was aspirated and cells were washed twice using pre-warmed PBS buffer. Next, 150 µL MTT reagent diluted in culture medium was added on top of the surfaces to be tested and incubated at 37 °C for 4 hours in a dark. MTT reagent was then removed from the culture medium followed by addition of 200 µL of DMSO to dissolve the purple crystals, and the plates were kept on shaking using a rotary shaker at 100 rpm for 15 min. For the reading, 200 µL solution was taken from each well and transferred to 96-well plates. Finally, the absorbances were measured at 570 nm. The DMEM medium was used as a blank. Cell viability was calculated using equation 1 and the % cell viability results are presented below:

$$\text{Cell viability}=\frac{Absorbance\ (Sample)-Absorbance\ (Blank)}{Absorbance\ (Control)-Absorbance\ (Blank)}\ \text{x}\ 100 \qquad (1)$$

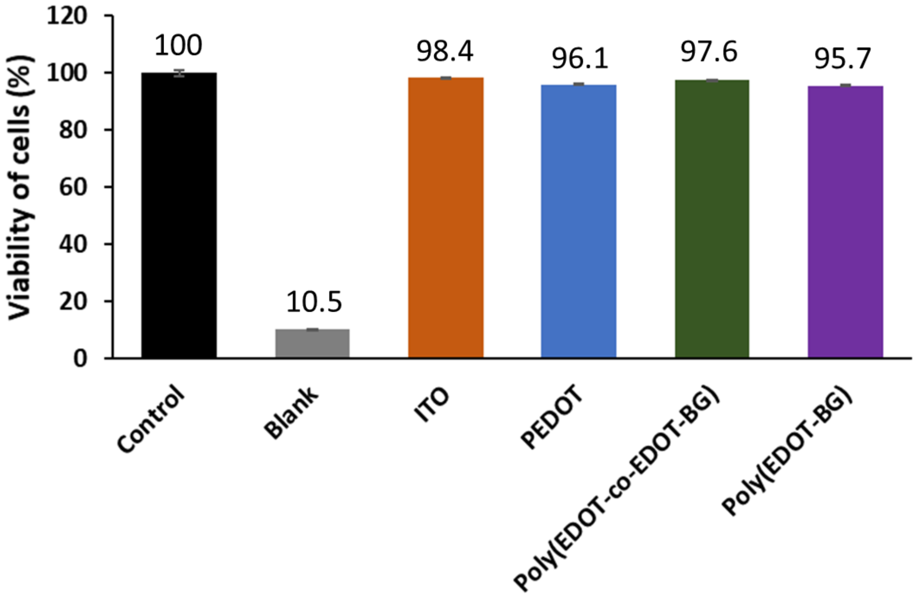

Figure S2. MTT viability assay graph showing viability (%) of HEK-293T cells cultured on different substrates for 24 hours.

Antifouling property studies

A 50 µL of *E. coli* at a concentration of 10^6 cells/mL was pipetted onto each substrate. The sample was incubated at room temperature for 2 hours. Later, the sample was washed with PBS and stained with live and dead bacterial staining kit. The number of cells was determined with a (IX71, Olympus, Japan) mounted on Olympus IX81 fluorescent microscopy (Olympus, Japan) with 10X and 40x objective lens. Three separate samples were analyzed for each substrate.

1 Spectra of the Compounds

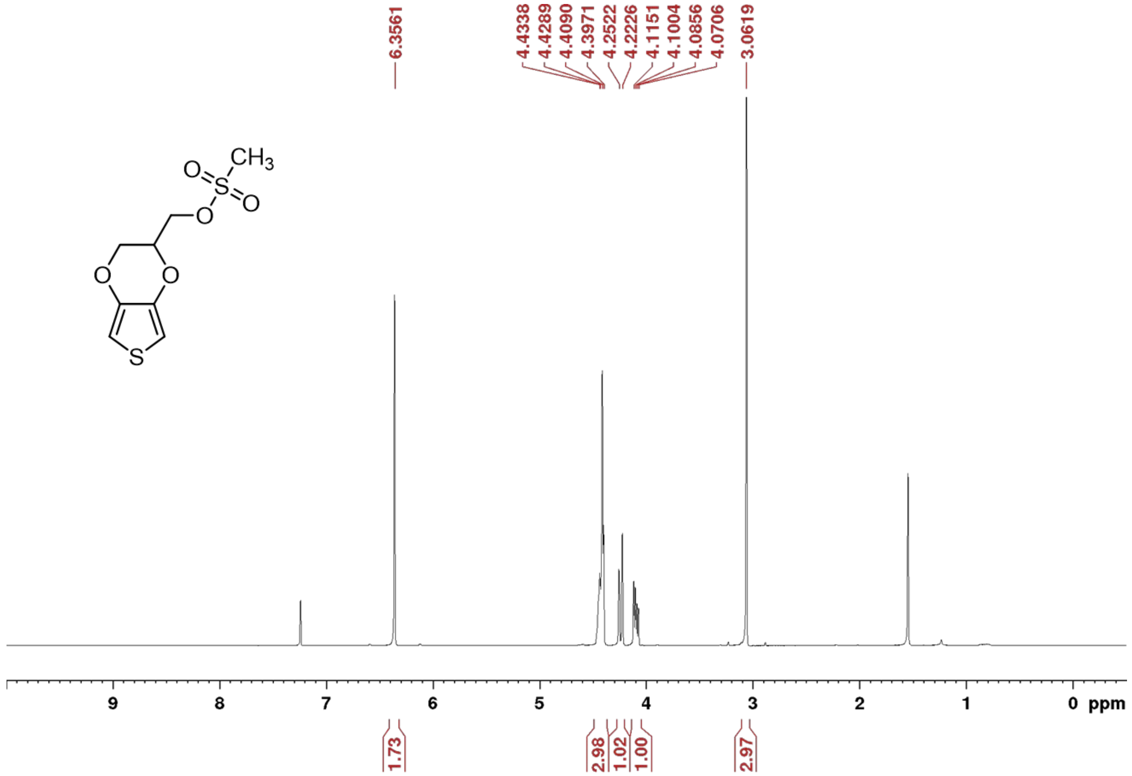

Figure S3. ^{1}H NMR of EDOT-O-SO_2CH_3 (400 MHz, $CDCl_3$)

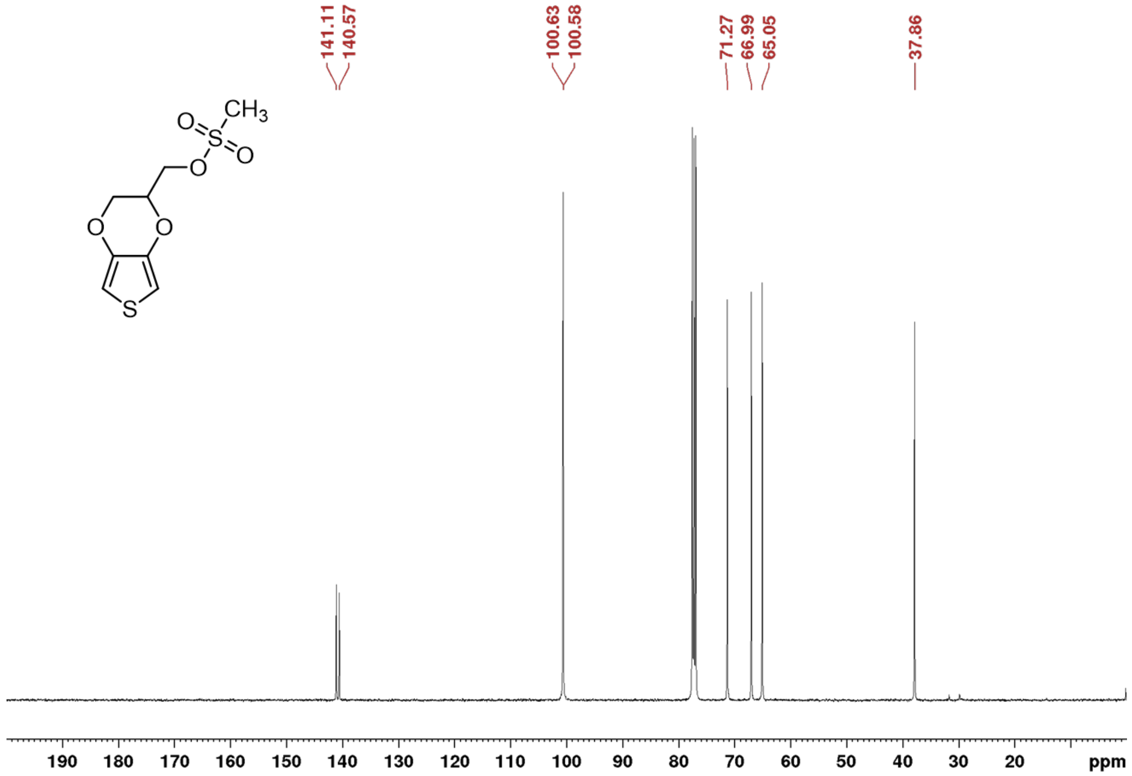

Figure S4. ^{13}C NMR of EDOT-SO_2CH_3 (100 MHz, $CDCl_3$)

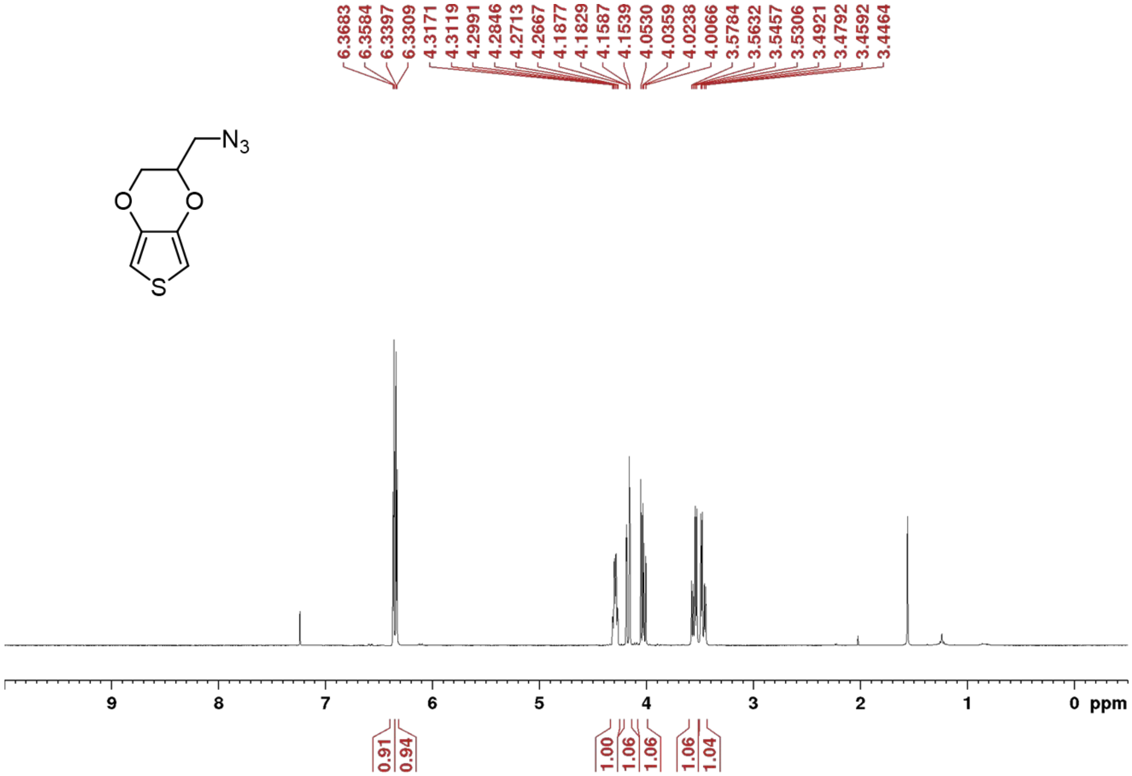

Figure S5. ^{1}H NMR of EDOT-N_3 (400 MHz, $CDCl_3$)

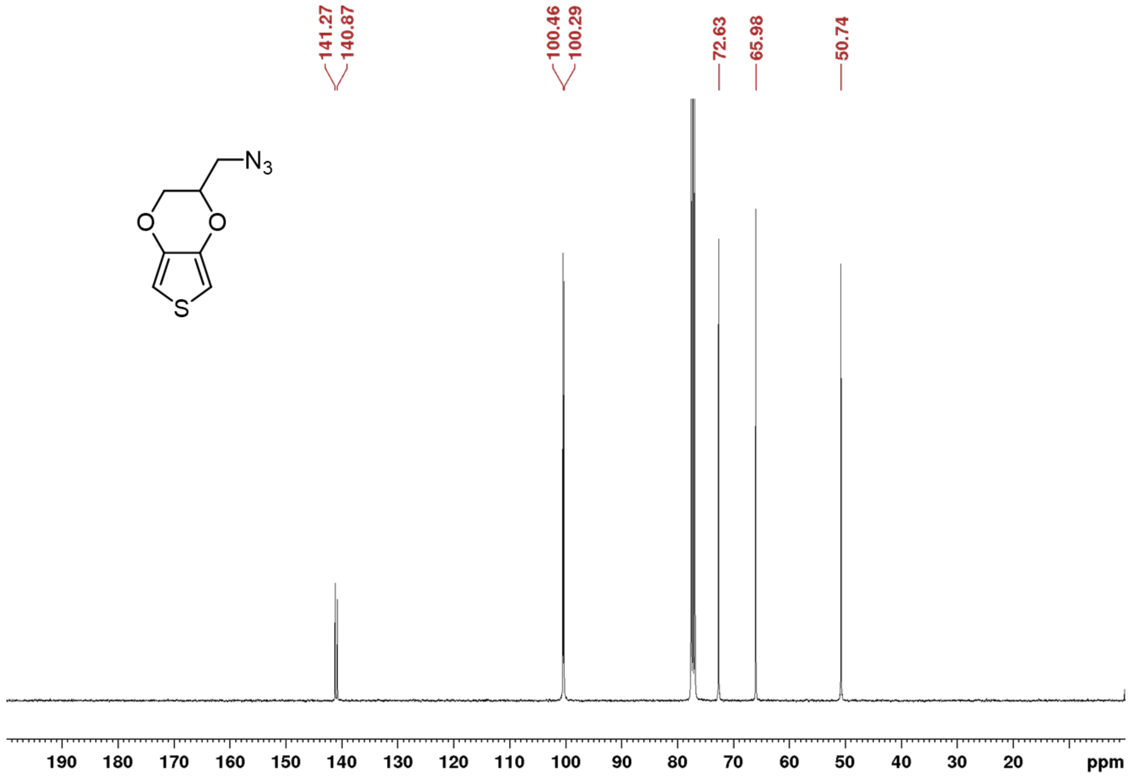

Figure S6. ^{13}C NMR of EDOT-N_3 (100 MHz, $CDCl_3$)

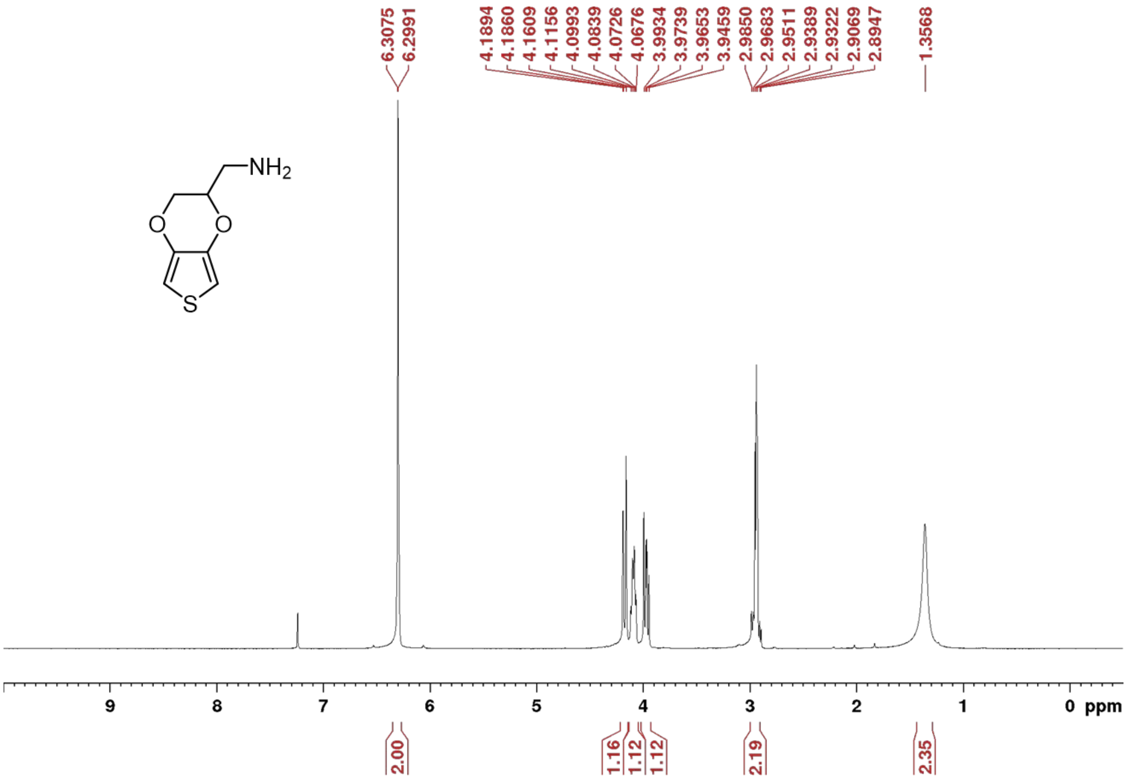

Figure S7. ^{1}H NMR of EDOT-NH_2 (400 MHz, $CDCl_3$)

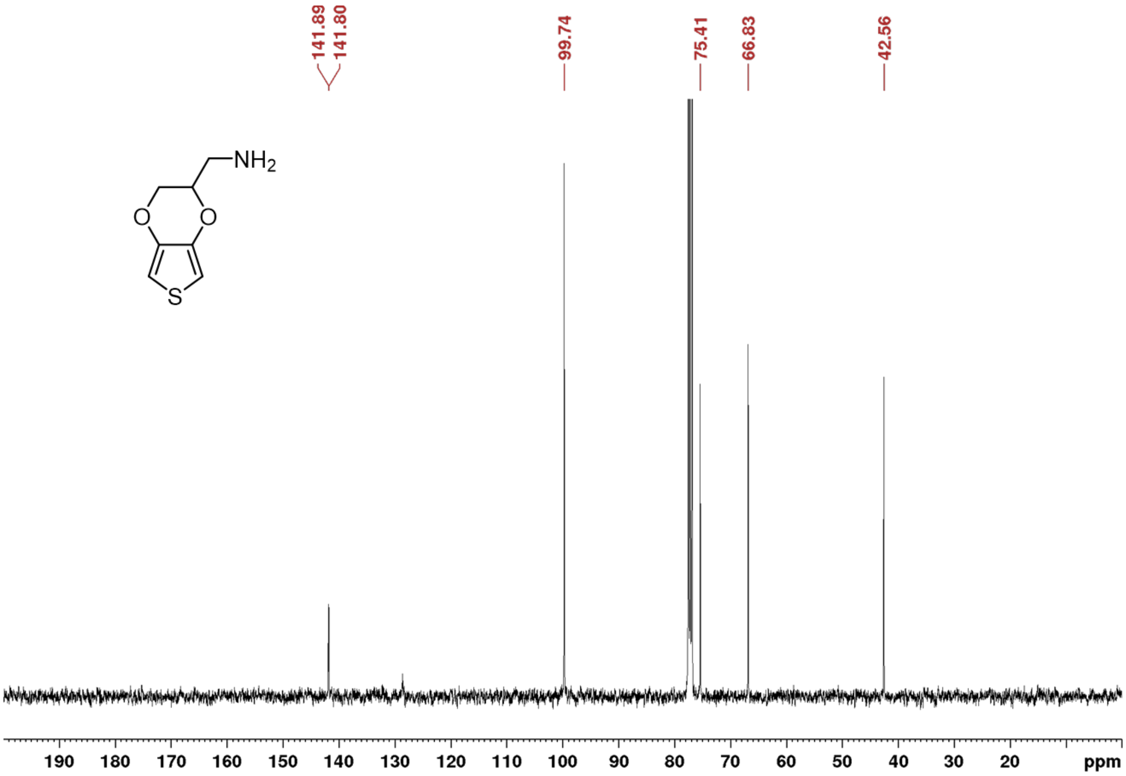

Figure S8. ^{13}C NMR of EDOT-NH_2 (100 MHz, $CDCl_3$)

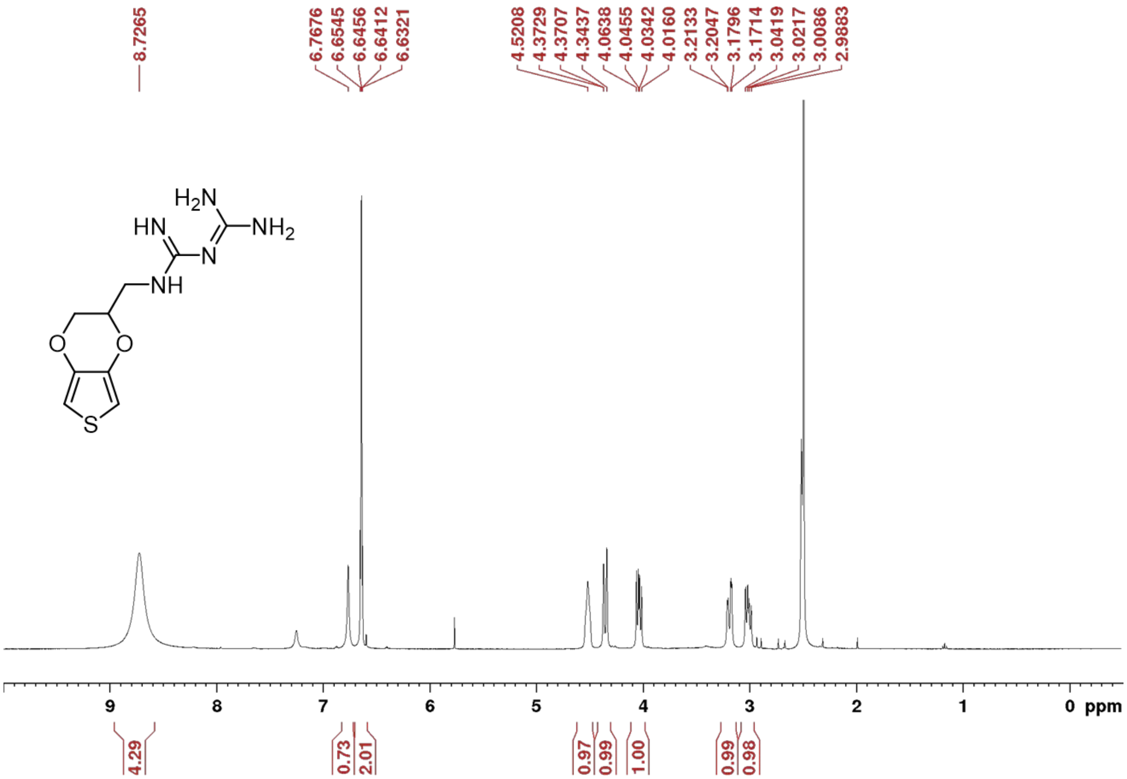

Figure S9. ^{1}H NMR of EDOT-BG (400 MHz, DMSO-d_6)

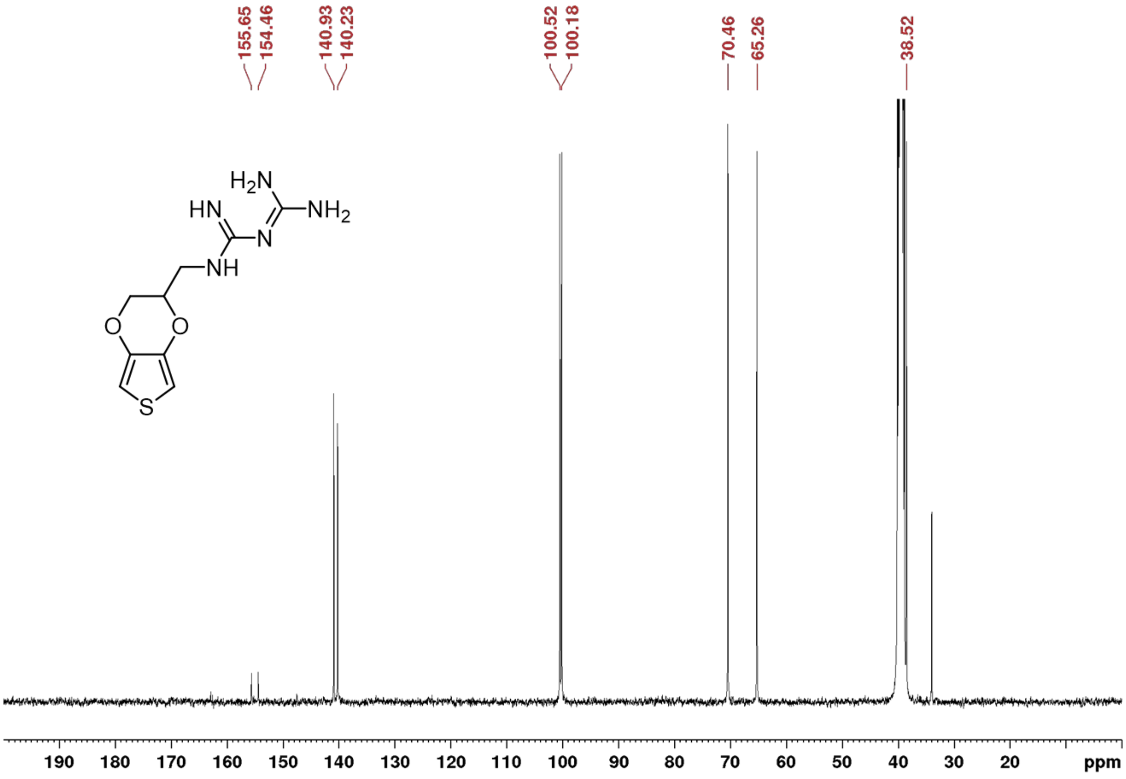

Figure S10. ^{13}C NMR of EDOT-BG (400 MHz, DMSO-d_6)

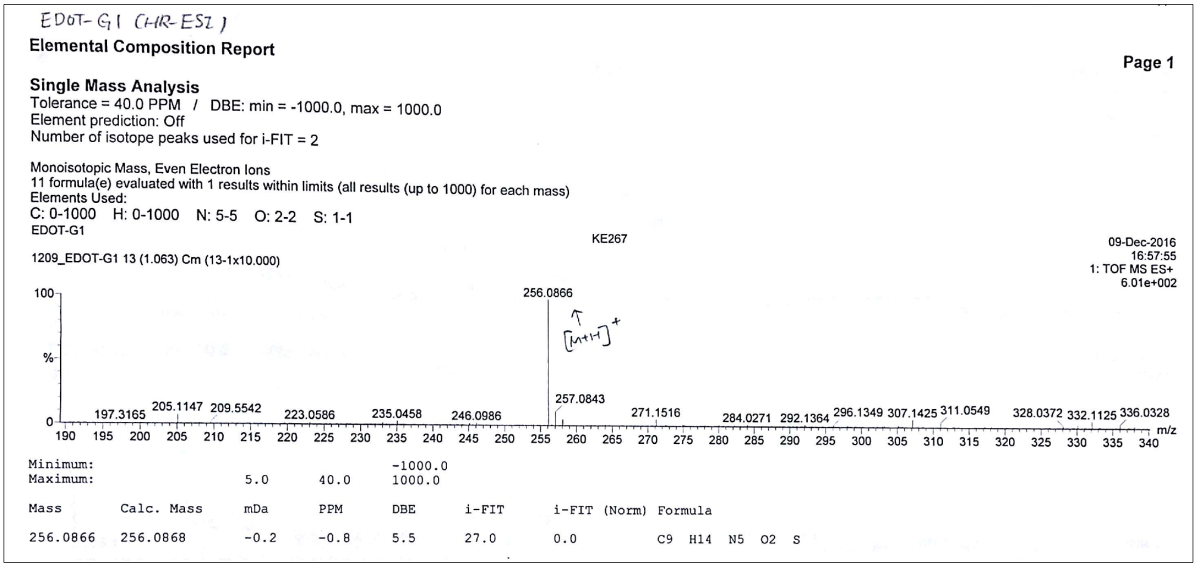

Figure S11. MS of EDOT-BG

Reference

Chen, C. H. and Luo, S. C. (2015). Tuning Surface Charge and Morphology for the Efficient Detection of Dopamine under the Interferences of Uric Acid, Ascorbic Acid, and Protein Adsorption. *ACS Appl. Mater. & Interfaces,* 7, 21931-21938. https://doi.org/10.1021/acsami.5b06526
